# Supplementary material for: Mechanically induced pyroptosis enhances cardiosphere oxidative stress resistance and metabolism for myocardial infarction therapy
Source: Nat Commun. 2023 Oct 2;14:6148. doi: 10.1038/s41467-023-41700-0 (PMC10545739; doi:10.1038/s41467-023-41700-0)
Supplement: Supplementary file 1 — Supplementary Information [file 41467_2023_41700_MOESM1_ESM.pdf]

## Supplementary information

# Mechanically induced pyroptosis enhances cardiosphere oxidative stress resistance and metabolism for myocardial infarction therapy

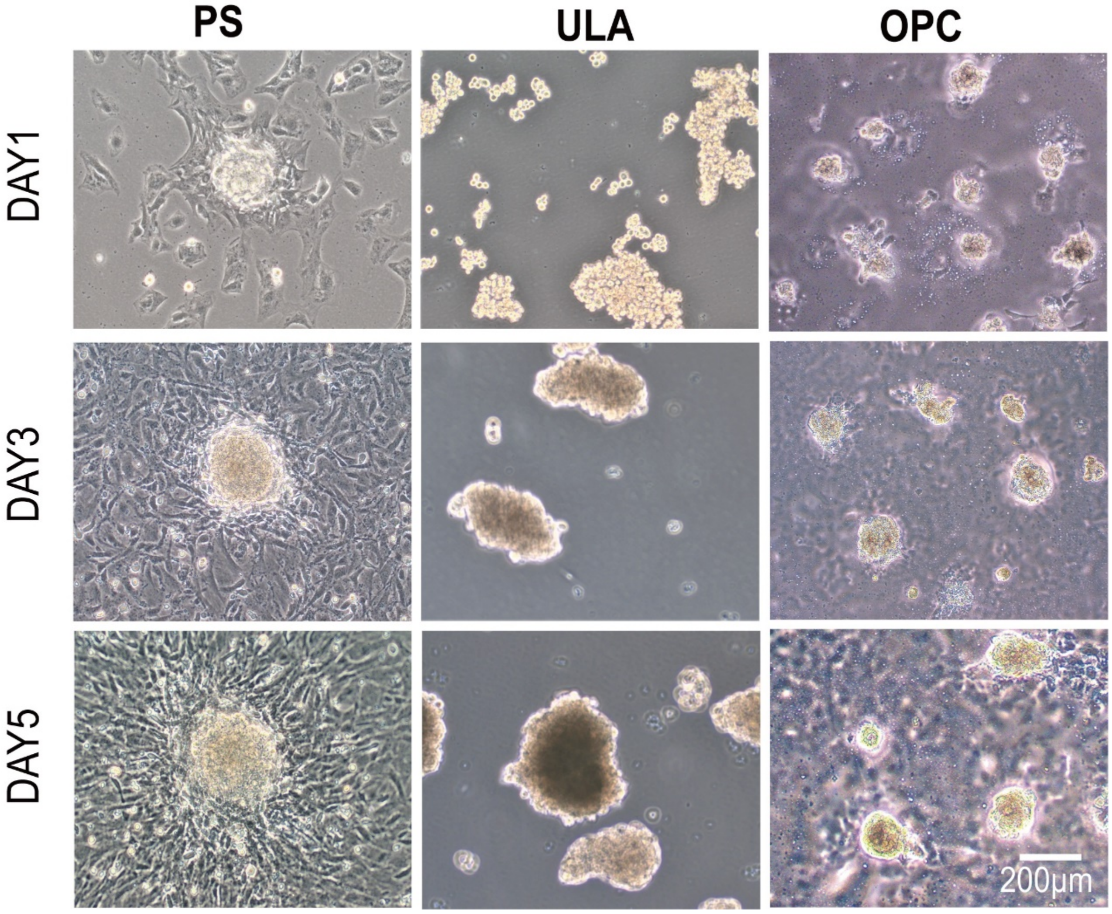


**Supplementary Fig. 1. Morphological changes of CSps on the PS, ULA, and OPC substrates in high magnification fields.** The formation of CSps on the PS substrate, the ULA substrate, and the OPC substrate were observed and recorded on day 1, 3 and 5, respectively.

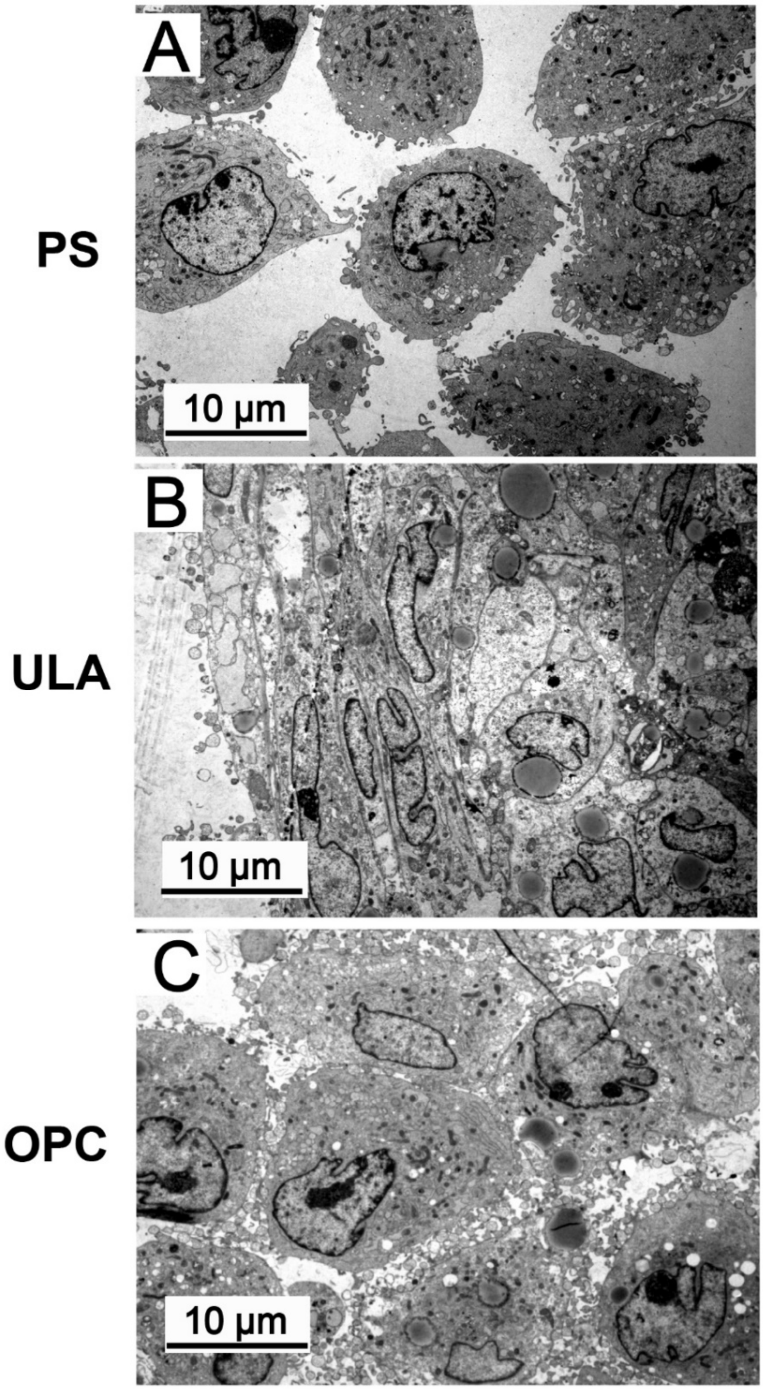


**Supplementary Fig. 2. TEM analysis results of the cellular ultrastructure in different groups.** The ultrastructure of CDCs from the PS group, the ULA group, and the OPC group are shown. Highly cell aggregation was observed in the ULA-CSps, while the cells in OPC-CSps remained certain cell-cell spaces and normal cell ultrastructure.

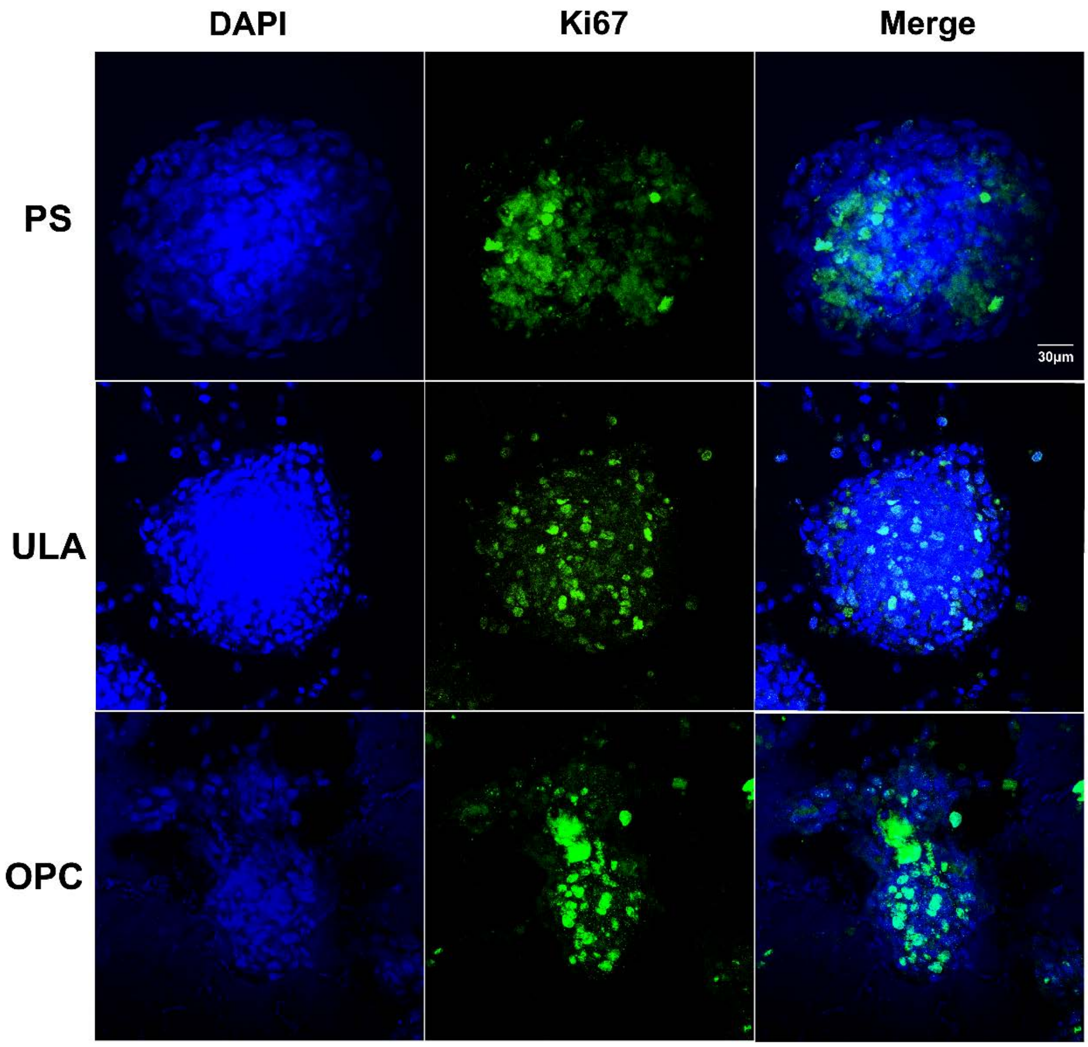


**Supplementary Fig. 3. Representative images of Ki67 immunofluorescence staining results of the CSps from each group following 3 days of cultivation.** CSps samples from each group were used for analysis. The cell nucleus was detected by staining with Dapi (blue), and the expression of anti-Ki67 antibodies (green) were co-stain to determine the proliferated cells in the CSps from each group.

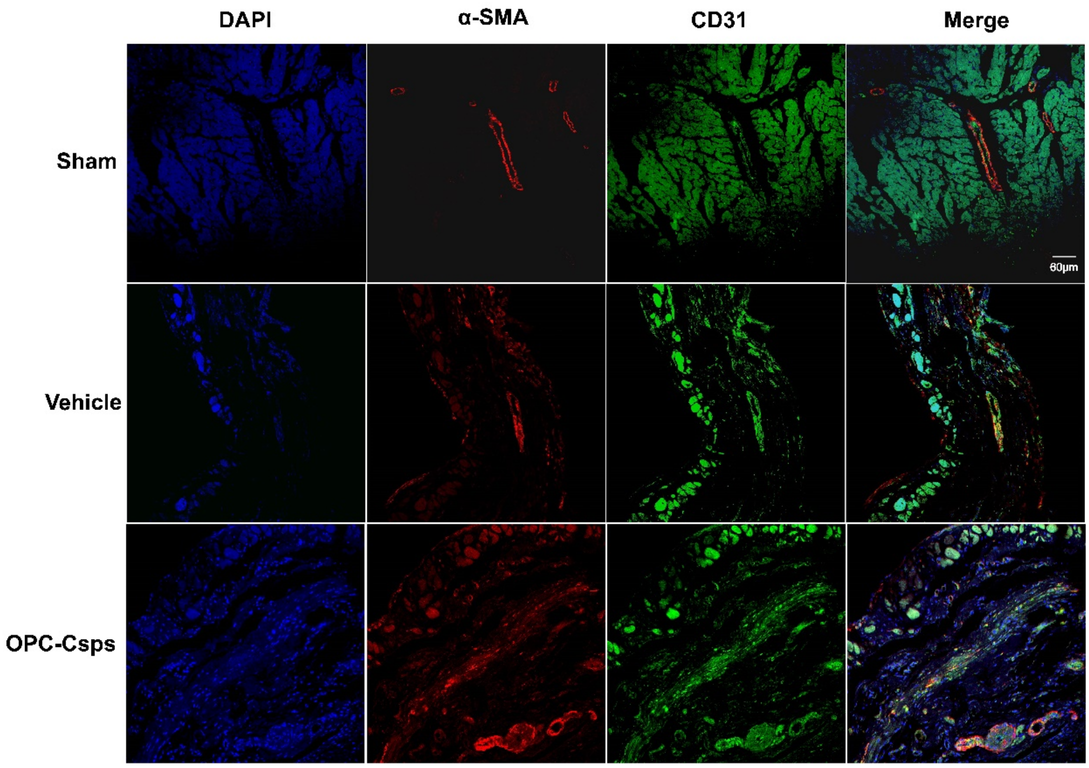


**Supplementary Fig. 4. Spilt channels images of the α-SMA and CD31 immunofluorescence staining results of Fig. 7b.** Paraffin sections of the hearts from each group were used for analysis. The cell nucleus was detected by staining with Dapi (blue), and the expression of anti-CD31 (endothelial cell marker) antibodies (green) and anti-α-SMA (smooth muscle cell marker) antibodies (red) were co-stain to determine the vascular distribution of the left ventricle area.

## Supplementary Table 1

| Supplementary Table 1: List of qRT-PCR primer sequences. | | |
|---|---|---|
| Gene | Primer Forward (5'-3') | Primer Reverse (5'-3') |
| GAPDH | AAGGTCGGAGTCAACGGATTT | AGATGATGACCCTTTTGGCTC |
| Caspase-1 | ATGGCCGACAAGGTCCTGAGG | GTGACATGATCGCACAGGTCTCG |
| Il-1β | GCAGGCAGTATCACTCATTGT | GGCTTTTTTGTTGTTCATCTC |
| Oct4 | AAGAGGATCACCTTGGGGTACA | CACCAGGGTCTCCGATTTG |
| Nanog | CTTGCCGTTGGGCTGACAT | GCTTTAGCTTGGGATTGCTAGAA |
| Sox2 | CTTCGCAGGGAGTTCTCAAAA | TTCCTTCCTTGTCTGTAACGGTC |
| VEGF | CGACAGAAGGGGAGCAGAAA | GCTGGCTTTGGTGAGGTTTG |
| HGF | CCTTCGAGCTATCGCGGTAA | GAATTTGTGCCGGTGTGGTG |
| IGF-1 | CAAAATGAGCGCACCTCCAA | CTTCAGCGGAGCACAGTACA |
| bFGF | GATCCCAAGCGGCTCTACTG | CCGTGACCGGTAAGTGTTGT |
| HK2 | CCAGCAGAACAGCCTAGACC | AGATGCCTTGAATCCCTTTG |
| LDHA | TTGTTGGGGTTGGTGC | TCCCTCTTGCTGACGG |
| PFKL | CCAGCCACCATCAGCAACAA | TGTCTGTCTTCATCTTCTCTGTCAT |
| CS | CCGTGCTCATGGACTTGGGCCTT | CCCCTGGCCCAACGTAGATGCTC |
| COXII | AGAAGCGAGGACCTGGGTTCA | ACACCTCTCCACCGATGACCTG |
| IDH2 | CCCATCACCATTGGCAGACAC | CCTCCGGCAGGGAAGTTATACA |
| SDHA | CTCTTTCCTACCCGCTCACATAC | TGTCATAGAAATGCCATCTCCAG |
| MDH2 | TTGCATCATTTCCAACCCAG | TGAAAACTTCGGCTGTGATGG |
