# Supplementary material for: Mechanically induced pyroptosis enhances cardiosphere oxidative stress resistance and metabolism for myocardial infarction therapy
Source: Nat Commun. 2023 Oct 2;14:6148. doi: 10.1038/s41467-023-41700-0 (PMC10545739; doi:10.1038/s41467-023-41700-0)
Supplement: Supplementary file 3 — Reporting Summary [file 41467_2023_41700_MOESM3_ESM.pdf]

nature portfolio

Corresponding author(s): Zheng Wu & Jianhua Zhang

Last updated by author(s): Aug 16, 2023

Reporting Summary

Nature Portfolio wishes to improve the reproducibility of the work that we publish. This form provides structure for consistency and transparency in reporting. For further information on Nature Portfolio policies, see our Editorial Policies and the Editorial Policy Checklist.

Statistics

For all statistical analyses, confirm that the following items are present in the figure legend, table legend, main text, or Methods section.

n/a	Confirmed	
☐	☒	The exact sample size (*n*) for each experimental group/condition, given as a discrete number and unit of measurement
☐	☒	A statement on whether measurements were taken from distinct samples or whether the same sample was measured repeatedly
☐	☒	The statistical test(s) used AND whether they are one- or two-sided<br>*Only common tests should be described solely by name; describe more complex techniques in the Methods section.*
☒	☐	A description of all covariates tested
☐	☒	A description of any assumptions or corrections, such as tests of normality and adjustment for multiple comparisons
☐	☒	A full description of the statistical parameters including central tendency (e.g. means) or other basic estimates (e.g. regression coefficient) AND variation (e.g. standard deviation) or associated estimates of uncertainty (e.g. confidence intervals)
☐	☒	For null hypothesis testing, the test statistic (e.g. *F*, *t*, *r*) with confidence intervals, effect sizes, degrees of freedom and *P* value noted<br>*Give P values as exact values whenever suitable.*
☒	☐	For Bayesian analysis, information on the choice of priors and Markov chain Monte Carlo settings
☒	☐	For hierarchical and complex designs, identification of the appropriate level for tests and full reporting of outcomes
☒	☐	Estimates of effect sizes (e.g. Cohen's *d*, Pearson's *r*), indicating how they were calculated

Our web collection on statistics for biologists contains articles on many of the points above.

Software and code

Policy information about availability of computer code

Data collection	Flow cytometry was collected with a BD FACS Canto (BD Bioscience). qRT-PCR was run using a Mini Cycler PCR instrument (Bio-rad Laboratories). Cell ultrastructure was visualized with transmission electron microscope (JEOL). Sequencing was performed on the Illumina NovaSeq 6000 platform.Transthoracic echocardiography was performed using a Vevo 2100. An inverted microscope (Olympus IX71) and laser confocal scanning microscope (Olympus, FV3000) were used for observing the morphology of CSps and the stained images, respectively.
Data analysis	Flow cytometry data was analyzed by Flow Jo software (BD Bioscience, V 10.8.1). Image J software V1.8.0.112 was used for section quantification and cell morphology measurements. Olympus FV31S-SW software and Bruker Molecular Imaging Software (IB5438150 Rev. B 12/12) were both used for analysis. Statistical analysis was performed using GraphPad Prism version 9.0 software. Significant differentially expressed genes (DEGs) among cells cultured on the PS, ULA, and OPC substrates were evaluated using DESeq (1.28.0). Kobas (3.0) was used for Kyoto Encyclopedia of Genes and Genomes (KEGG) pathway enrichment analysis. Gene set enrichment analysis (GSEA) was carried out using GSEA v.4.2.3.

For manuscripts utilizing custom algorithms or software that are central to the research but not yet described in published literature, software must be made available to editors and reviewers. We strongly encourage code deposition in a community repository (e.g. GitHub). See the Nature Portfolio guidelines for submitting code & software for further information.

Data

Policy information about availability of data

All manuscripts must include a data availability statement. This statement should provide the following information, where applicable:

- Accession codes, unique identifiers, or web links for publicly available datasets
- A description of any restrictions on data availability
- For clinical datasets or third party data, please ensure that the statement adheres to our policy

Source data are provided with this paper. The experimental data generated in this study are provided in the Source Data file. The RNAseq data from CDCs cultured on the PS, ULA, and OPC substrates used in this study are available in the GEO database under accession code GSE223508 [https://www.ncbi.nlm.nih.gov/geo/query/acc.cgi?acc=GSE223508].

Human research participants

Policy information about studies involving human research participants and Sex and Gender in Research.

Reporting on sex and gender	N/A
Population characteristics	N/A
Recruitment	N/A
Ethics oversight	N/A

Note that full information on the approval of the study protocol must also be provided in the manuscript.

Field-specific reporting

Please select the one below that is the best fit for your research. If you are not sure, read the appropriate sections before making your selection.

☒ Life sciences ☐ Behavioural & social sciences ☐ Ecological, evolutionary & environmental sciences

For a reference copy of the document with all sections, see nature.com/documents/nr-reporting-summary-flat.pdf

Life sciences study design

All studies must disclose on these points even when the disclosure is negative.

Sample size	Power analysis was used to estimate the sample size of the experiments. A preliminary experiment was performed with a control group and a model group, mean value, standard deviation, and monitor parameters (e.g. LVEF% in the in vivo experiment) were collected. Then power analysis was performed to estimate the sample size with two-side Student's t test or one-way ANOVA, and the significance level of 0.05 and statistical power of 0.9 were used. By following this procedure, we can get that the estimated sample size, and the final sample size were further adjusted according to the practical feasibility, ethical factors and resource availability. The minimum sample size of the in vivo experiment was calculated as 6.5, and finally 8 animals were used in each group considering the uncontrollable factors. For the in vitro experiments sample size was performed with a minimum of 3 independent experiments.
Data exclusions	No data was excluded.
Replication	Each experimental protocol was successfully replicated a minimum of three times.
Randomization	All samples were randomly selected and assigned to each group for analysis.
Blinding	For in vivo animal experiments, invesigators were blined to surgical procedures, injections, and analysis. Blinding was not feasible for in vitro experiments since implementation needed treatment groups to be revealed to the researcher as part of the study design for accurate execution.Recording the outcomes assessments was done with the utmost objectivity at all times. Results were trustworthy, there were numerous assessments of the results, high levels of agreement among the various assessors, and instrumentation was used that allowed for objective quantification. Together, these steps reduce the possibility of bias affecting data processing and interpretation.

Reporting for specific materials, systems and methods

We require information from authors about some types of materials, experimental systems and methods used in many studies. Here, indicate whether each material, system or method listed is relevant to your study. If you are not sure if a list item applies to your research, read the appropriate section before selecting a response.

Materials & experimental systems

n/a	Involved in the study
☐	☒ Antibodies
☒	☐ Eukaryotic cell lines
☒	☐ Palaeontology and archaeology
☐	☒ Animals and other organisms
☒	☐ Clinical data
☒	☐ Dual use research of concern

Methods

n/a	Involved in the study
☒	☐ ChIP-seq
☐	☒ Flow cytometry
☒	☐ MRI-based neuroimaging

Antibodies

Antibodies used

CD31 Servicebio Cat#: GB11063-3
CD34 Abcam Cat#: ab81289
CD90 Abcam Cat#: ab225
CD105 Abcam Cat#: ab156756
Sca-1 Abcam Cat#: ab51317
KDR Santacruz Cat#: sc6251
Alexa Fluor 488 goat anti-mouse IgG Abcam Cat#: ab150113
Alexa Fluor 488 goat anti-rabbit IgG Abcam Cat#: ab150077
Alexa Fluor 594 goat anti-rabbit IgG Abcam Cat#: ab150080
Alexa Fluor 594 goat anti-mouse IgG Abcam Cat#: ab150116
HRP-labeled Anti-Rabbit IgG antibodies Cell Signal Technology Cat#: 7074
HRP-labeled Anti-Rat IgG antibodies anti-mouse Cell Signal Technology Cat#: 7076
caspase-1 Abcam Cat#: ab1872
β-actin Abcam Cat#: ab8226
CD68 ZhengNeng Cat#: 360018
Cardiac troponin T (cTnT) Abcam Cat#: ab209813
Smooth muscle alpha-actin (α-SMA) SAB Cat#: 41550

Validation

1 CD31 Servicebio Cat#: GB11063-3
website https://www.servicebio.cn/search-result?search=GB11063-3
Species human, mouse, rat Applications: IHC/IF
Reference?①Wu, J. et al.Immunity-and-matrix-regulatory cells derived from human embryonic stem cells safely and effectively treat mouse lung injury and fibrosis.Cell research30,794-809(2020).PMID 32546764Application: IFIF 46.297
② Zeng, Y. et alAnti-angiogenesis triggers exosomes release from endothelial cells to promote tumor vasculogenesis.J Extracell Vesicles8,1629865-1629865(2019).PMID 31258881Application: IHCIF 25.841

2 CD34 Abcam Cat#:ab81289
Website:https://www.abcam.cn/products/primary-antibodies/cd34-antibody-ep373y-ab81289.html
Species Rabbit Applications: WB, IHC-P, ICC/IF, IP, IHC-Fr, Flow Cyt (Intra)
Reference?①Lefort S et al. De novo and cell line models of human mammary cell transformation reveal an essential role for Yb-1 in multiple stages of human breast cancer. Cell Death Differ 29:54-64 (2022).
② You D et al. miR-223-3p inhibits the progression of atherosclerosis via down-regulating the activation of MEK1/ERK1/2 in macrophages. Aging (Albany NY) 14:1865-1878 (2022).

3 CD90 Abcam Cat#: ab225
Website:https://www.abcam.cn/products/primary-antibodies/cd90--thy1-antibody-mrc-ox-7-hematopoietic-stem-cell-marker-ab225.html
Species Mouse Applications: ICC, WB, Flow Cyt (Intra)
Reference?① Zhu K et al. Effect of lentivirus-mediated growth and differentiation factor-5 transfection on differentiation of rabbit nucleus pulposus mesenchymal stem cells. Eur J Med Res 27:5 (2022).
② Zhang Y et al. Sirt5-mediated desuccinylation of OPTN protects retinal ganglion cells from autophagic flux blockade in diabetic retinopathy. Cell Death Discov 8:63 (2022).

4 CD105 Abcam Cat#: ab156756
Website:https://www.abcam.cn/products/primary-antibodies/cd105-antibody-oti8a1-ab156756.html
Species Mouse Applications: WB, ICC/IF, Flow Cyt (Intra)
Reference?① Niu W et al. Platelet-Derived Growth Factor Stimulated Migration of Bone Marrow Mesenchymal Stem Cells into an Injectable Gelatin-Hydroxyphenyl Propionic Acid Matrix. Biomedicines 9:N/A (2021).
② Guillamat-Prats R et al. Alveolar Type II Cells or Mesenchymal Stem Cells: Comparison of Two Different Cell Therapies for the Treatment of Acute Lung Injury in Rats. Cells 9:N/A (2020).

5 Sca-1 Abcam Cat#: ab51317
Website:https://www.abcam.cn/products/primary-antibodies/ly-6ae-sca-1-antibody-e13-161-7-hematopoietic-stem-cell-marker-ab51317.html
Species Rat Applications: Flow Cyt, ICC/IF
Reference

① Figeac F et al. Impaired Bone Fracture Healing in Type 2 Diabetes Is Caused by Defective Functions of Skeletal Progenitor Cells. Stem Cells 40:149-164 (2022).
② Zhou X et al. SM22α-lineage niche cells regulate intramembranous bone regeneration via PDGFRβ-triggered hydrogen sulfide production. Cell Rep 39:110750 (2022).

6 KDR Santacruz Cat#: sc6251
Website https://www.scbt.com/p/vegfr2-antibody-a-3?requestFrom=search
Species Rat Applications: Flow Cyt, ICC/IF
Reference?① Di Nisio V, Rossi G, Chiominto A, Pompili E, Cecconi S. Do Aging and Parity Affect VEGF-A/VEGFR Content and Signaling in the Ovary?-A Mouse Model Study. Int J Mol Sci. 2023 Feb 7;24(4):3318.
② Fiorimanti MR, Cristofolini AL, Moreira-Espinoza MJ, Rabaglino MB, Barbeito CG, Merkis CI. Placental vascularization in middle and late gestation in the pig. Reprod Fertil. 2022 Mar 8;3(1):57-66.

7 Alexa Fluor 488 goat anti-mouse lgG Abcam Cat#: ab150113
Website:https://www.abcam.cn/products/secondary-antibodies/goat-mouse-igg-hl-alexa-fluor-488-ab150113.html
Species Goat Applications: IHC-Fr, ICC/IF, ELISA, Flow Cyt, IHC-P
Reference:① Kraljević M et al. Prognostic and predictive significance of VEGF, CD31, and Ang-1 in patients with metastatic clear cell renal cell carcinoma treated with first-line sunitinib. Biomol Biomed 23:161-169 (2023).
② Asif H et al. Title: β3 Adrenergic Receptor Signaling in the Human Myometrium. Reprod Sci 30:124-134 (2023).

8 Alexa Fluor 488 goat anti-rabbit lgG Abcam Cat#: ab150077
Website https://www.abcam.cn/products/secondary-antibodies/goat-rabbit-igg-hl-alexa-fluor-488-ab150077.html
Species Goat Applications: ICC/IF, Flow Cyt, IHC-P, ELISA, IHC-Fr
Rerference?① He XF et al. Inhibition of phosphorylated calcium/calmodulin-dependent protein kinase IIα relieves streptozotocin-induced diabetic neuropathic pain through regulation of P2X3 receptor in dorsal root ganglia. Purinergic Signal 19:99-111 (2023).
② Jin L et al. ROS-responsive 18β-glycyrrhetic acid-conjugated polymeric nanoparticles mediate neuroprotection in ischemic stroke through HMGB1 inhibition and microglia polarization regulation. Bioact Mater 19:38-49 (2023).

9 Alexa Fluor 594 goat anti-rabbit lgG Abcam Cat#: ab150080
Website: https://www.abcam.cn/products/secondary-antibodies/goat-rabbit-igg-hl-alexa-fluor-594-ab150080.html
Species: Goat Application:IHC-Fr, ICC/IF, ELISA, IHC-P, Flow Cyt
Reference?① Asif H et al. Title: β3 Adrenergic Receptor Signaling in the Human Myometrium. Reprod Sci 30:124-134 (2023).
② Ouyang L et al. Puerarin@Chitosan composite for infected bone repair through mimicking the bio-functions of antimicrobial peptides. Bioact Mater 21:520-530 (2023).
10 Alexa Fluor 594 goat anti-mouse lgG Abcam Cat#: ab150116
Website https://www.abcam.cn/products/secondary-antibodies/goat-mouse-igg-hl-alexa-fluor-594-ab150116.html
Species: Goat Application: IHC-Fr, ICC/IF, ELISA, IHC-P, Flow Cyt
Reference:①Chen Z et al. Spinal CircKcnk9 Regulates Chronic Visceral Hypersensitivity of Irritable Bowel Syndrome. J Pain 24:463-477 (2023).
② Scalzone A et al. An In Vitro Engineered Osteochondral Model as Tool to Study Osteoarthritis Environment. Adv Healthc Mater 12:e2202030 (2023).

11 HRP-labeled Anti-Rabbit lgG antibodies Cell Signal Technology Cat#: 7074
Website:https://www.cellsignal.cn/products/secondary-antibodies/anti-rabbit-igg-hrp-linked-antibody/7074?site-search-type=Products&N=4294956287&Ntt=7074&fromPage=plp&_requestid=284743
Species: Goat Application: WB, IP ,IHC,,eCLIP, IF, Flow Cyt
Rerference: ①Qin XD, Yang TQ, Zeng JH, Cai HB, Qi SH, Jiang JJ, Cheng Y, Xu LS, Bu F. Overexpression of mitogen-activated protein kinase phosphatase-1 in endothelial cells reduces blood-brain barrier injury in a mouse model of ischemic stroke. Neural Regen Res. 2023 Aug;18(8):1743-1749.
② Li XH, Zhu HC, Cui XM, Wang W, Yang L, Wang LB, Hu NW, Duan DX. Death-associated protein kinase 1 is associated with cognitive dysfunction in major depressive disorder. Neural Regen Res. 2023 Aug;18(8):1795-1801.

12 HRP-labeled Anti-Rat lgG antibodies anti-mouse Cell Signal Technology Cat#: 7076
Website:https://www.cellsignal.cn/products/secondary-antibodies/anti-mouse-igg-hrp-linked-antibody/7076?site-search-type=Products&N=4294956287&Ntt=7076&fromPage=plp&_requestid=295340
Species: horse Application: WB, IP ,IHC, eCLIP, IF, Flow Cyt
Rerference:①Ryu W, Park CW, Kim J, Lee H, Chung H. The Bcl-2/Bcl-xL Inhibitor ABT-263 Attenuates Retinal Degeneration by Selectively Inducing Apoptosis in Senescent Retinal Pigment Epithelial Cells. Mol Cells. 2023 Jul 31;46(7):420-429.
② Mourkioti I, Polyzou A, Veroutis D, Theocharous G, Lagopati N, Gentile E, Stravokefalou V, Thanos DF, Havaki S, Kletsas D, Panaretakis T, Logothetis CJ, Stellas D, Petty R, Blandino G, Papaspyropoulos A, Gorgoulis VG. A GATA2-CDC6 axis modulates androgen receptor blockade-induced senescence in prostate cancer. J Exp Clin Cancer Res. 2023 Jul 29;42(1):187.

13 caspase-1 Abcam Cat#:ab1872
Website https://www.abcam.cn/products/primary-antibodies/caspase-1-antibody-ab1872.html
Note:Anti-Caspase-1 antibody is no longer available on the abcam platform (ab1872)
Species: rabbit Application: IHC-P, WB, ICC/IF Rerference:① Harris J, Hartman M, Roche C, Zeng SG, O'Shea A, Sharp FA, Lambe EM, Creagh EM, Golenbock DT, Tschopp J, Kornfeld H, Fitzgerald KA, Lavelle EC. Autophagy controls IL-1beta secretion by targeting pro-IL-1beta for degradation. J Biol Chem. 2011 Mar 18;286(11):9587-97.

14 B-actin Abcam Cat#:ab8226
Website https://www.abcam.cn/products/primary-antibodies/beta-actin-antibody-mabcam-8226-loading-control-ab8226.html
Species: mouse Application: ICC/IF, IHC-P, WB
Reference:①Wang S et al. Knockdown of circ_0004585 enhances the chemosensitivity of colorectal cancer cells to 5-fluorouracil via the miR-874-3p/CCND1 axis. Histol Histopathol 38:99-112 (2023).
② Fang M et al. MicroRNA-29b regulates pyroptosis involving calcific aortic valve disease through the STAT3/SOCS1 pathway. Int J Cardiol 371:319-328 (2023).

15 CD68 ZhengNeng Cat#: 360018
Website: http://www.zen-bio.cn/Private/Files/6380402795144781175133331 08.pdf
Species: rabbit Application: IHC-F,IHC-P,ICC/IF
Reference: Lin C, He Y, Feng Q, Xu K, Chen Z, Tao B, Li X, Xia Z, Jiang H, Cai K. Self-renewal or quiescence? Orchestrating the fate of mesenchymal stem cells by matrix viscoelasticity via PI3K/Akt-CDK1 pathway. Biomaterials. 2021 Dec;279:121235.

16 Cardiac troponin T(cTnT) Abcam Cat#: ab209813
Website:https://www.abcam.cn/products/primary-antibodies/cardiac-troponin-t-antibody-epr20266-ab209813.html
Species: rabbit Application: IHC-P, WB, Flow Cyt (Intra), ICC/IF
Reference:①Lv L et al. Pretreatment of Nicorandil Protects the Heart from Exhaustive Exercise-Induced Myocardial Injury in Rats. Evid Based Complement Alternat Med 2022:7550872 (2022).
② Shi J et al. Gut microbiota profiling revealed the regulating effects of salidroside on iron metabolism in diabetic mice. Front Endocrinol (Lausanne) 13:1014577 (2022).

17 Smooth muscle alpha-actin (a-SMA) SAB Cat#: 41550
Website https://www.sabbiotech.cn/g-15233-%CE%B1-SMA-Polyclonal-Antibody-41550.html
Species: rabbit Application: WB IHC ELISA I
Reference:①Gui Z, Suo C, Tao J, Wang Z, Zheng M, Fei S, Chen H, Sun L, Han Z, Ju X, Zhang H, Gu M, Tan R. Everolimus Alleviates Renal Allograft Interstitial Fibrosis by Inhibiting Epithelial-to-Mesenchymal Transition Not Only via Inducing Autophagy but Also via Stabilizing IκB-α. Front Immunol. 2022 Jan 24;12:753412.
②Liu Q, Wang R, Ma N, Wang C, Chen W. Telmisartan inhibits bladder smooth muscle fibrosis in neurogenic bladder rats. Exp Ther Med. 2022 Mar;23(3):216.

Animals and other research organisms

Policy information about studies involving animals; ARRIVE guidelines recommended for reporting animal research, and Sex and Gender in Research

Laboratory animals	3-month-old female Sprague Dawley rats(Vital River) ; 4-week-old female Sprague Dawley rats
Wild animals	No wild animals were used.
Reporting on sex	In order to identify the transplanted cells in the receipt animals, only 3-month-old female rats were used for established myocardium infarction animal model, and 4-week-old male rats were used for isolating primary cells and preparing for transplantation, so the therapeutic effect of transplanted CSps to MI rats were only evaluated in female animals in this study.
Field-collected samples	No filed-collected samples were used.
Ethics oversight	All animal studies were performed in accordance with the ethical guidelines of the National Guide for the Care and Use of Laboratory Animals and approved by Jinan University Animal Care and Use Committee (Approval numbers: I ACUC-20210113-06).

Note that full information on the approval of the study protocol must also be provided in the manuscript.

Flow Cytometry

Plots

Confirm that:

☒ The axis labels state the marker and fluorochrome used (e.g. CD4-FITC).

☒ The axis scales are clearly visible. Include numbers along axes only for bottom left plot of group (a 'group' is an analysis of identical markers).

☒ All plots are contour plots with outliers or pseudocolor plots.

☒ A numerical value for number of cells or percentage (with statistics) is provided.

Methodology

Sample preparation	For cell analysis, cells were fixed in a 75% ethanol solution for 10 minutes at room temperature after being collected and then labeled with antibodies.
Instrument	BD FACSCanto
Software	Flow Jo software (V 10.8.1)
Cell population abundance	10,000 events per sample were recorded

Gating strategy

Gating was set based on the commercially available secondary antibody or isotype controls. The background was gated to 2% or less based on secondary control.

☒ Tick this box to confirm that a figure exemplifying the gating strategy is provided in the Supplementary Information.
